# RhCMV serostatus and vaccine adjuvant impact immunogenicity of RhCMV/SIV vaccines

**DOI:** 10.1038/s41598-020-71075-x

**Published:** 2020-08-20

**Authors:** W. L. William Chang, Jesse D. Deere, Hung T. Kieu, Luis D. Castillo, Kawthar Machmach, Xiaoying Shen, Georgia D. Tomaras, Barbara L. Shacklett, Peter A. Barry, Dennis J. Hartigan-O’Connor, Ellen E. Sparger

**Affiliations:** 1 Center for Immunology and Infectious Diseases, Department of Medical Microbiology and Immunology, School of Medicine, University of California, Davis, CA USA; 2 California National Primate Research Center, University of California, Davis, CA USA; 3 Department of Medical Microbiology and Immunology, School of Medicine, University of California, Davis, CA USA; 4 Department of Medicine and Epidemiology, School of Veterinary Medicine, University of California, Davis, CA USA; 5 Department of Pathology, Microbiology, and Immunology, School of Veterinary Medicine, University of California, Davis, CA USA; 6 Walter Reed Army Institute of Research, Silver Spring, MD USA; 7 Department of Surgery, Duke Human Vaccine Institute, Duke University Medical Center, Durham, NC USA; 8 Division of Infectious Diseases, Department of Medicine, School of Medicine, University of California Davis, Davis, CA USA

**Keywords:** Infectious diseases, Vaccines, Adjuvants

## Abstract

Rhesus cytomegalovirus (RhCMV) strain 68-1-vectored simian immunodeficiency virus (RhCMV/SIV) vaccines are associated with complete clearance of pathogenic SIV challenge virus, non-canonical major histocompatibility complex restriction, and absent antibody responses in recipients previously infected with wild-type RhCMV. This report presents the first investigation of RhCMV/SIV vaccines in RhCMV-seronegative macaques lacking anti-vector immunity. Fifty percent of rhesus macaques (RM) vaccinated with a combined RhCMV-Gag, -Env, and -Retanef (RTN) vaccine controlled pathogenic SIV challenge despite high peak viremia. However, kinetics of viral load control by vaccinated RM were considerably delayed compared to previous reports. Impact of a TLR5 agonist (flagellin; FliC) on vaccine efficacy and immunogenicity was also examined. An altered vaccine regimen containing an SIV Gag-FliC fusion antigen instead of Gag was significantly less immunogenic and resulted in reduced protection. Notably, RhCMV-Gag and RhCMV-Env vaccines elicited anti-Gag and anti-Env antibodies in RhCMV-seronegative RM, an unexpected contrast to vaccination of RhCMV-seropositive RM. These findings confirm that RhCMV-vectored SIV vaccines significantly protect against SIV pathogenesis. However, pre-existing vector immunity and a pro-inflammatory vaccine adjuvant may influence RhCMV/SIV vaccine immunogenicity and efficacy. Future investigation of the impact of pre-existing anti-vector immune responses on protective immunity conferred by this vaccine platform is warranted.

## Introduction

Development of an efficacious human immunodeficiency virus (HIV-1) vaccine remains a high-priority goal. The only HIV-1 vaccine showing efficacy in human clinical trials remains the RV144 ALVAC-HIV (vCP1521) and AIDSVAX^®^ B/E regimen, which is based on a recombinant canarypox vector boosted by a recombinant glycoprotein gp120 subunit^1^. This vaccine was associated with 31.2% protection against HIV acquisition, with envelope (Env)-binding antibodies capable of antibody-dependent cytotoxicity implicated as an immune correlate of protection^1,2^. These encouraging results propelled further development of antibody-based approaches using the nonhuman primate (NHP) simian immunodeficiency virus (SIV) model with the goal of further optimizing protection through generation of antibodies with broadly neutralizing and/or Fc-receptor-mediated effector activity. These approaches, based on a wide assortment of viral and DNA vectors and recombinant protein immunogens, have shown modest protection against acquisition of challenge virus^3–6^. However, a news release by NIH/NIAD (2020) revealed that the HVTN 702 human clinical trial testing a vaccine approach based on the RV144 regimen, adapted to the subtype Clade C, and conducted in southern Africa, did not recapitulate the efficacy observed for the RV144 trial. Vaccine induction of Env-binding antibodies of sufficient potency and breadth to confer absolute protection against acquisition of divergent challenge viruses remains a significant challenge.

In contrast to antibody-based vaccines, cytotoxic T-cell-based vaccines focus on generation of HIV-specific T-cell responses of significant magnitude and breadth^3,7^. This approach has been tested with multiple viral vectors including adenovirus (Ad)5, Ad26, poxvirus, and rhabdovirus (rVSV), and all have demonstrated induction of strong antiviral T-cell responses in nonhuman primates. Notably, T-cell responses elicited by these viral vectors are biased towards a central memory phenotype^8^. A novel viral vectored- SIV vaccine encoding a near full-length SIV genome and based on the rhesus monkey rhadinovirus (RRV) was shown to induce both antibodies and T cell responses to SIV antigens^9^. One promising avenue for T-cell-based vaccines came from the unexpected results obtained using species-specific rhesus cytomegalovirus (RhCMV) as a vector for delivery of critical viral antigens (Gag, Retanef, Env, and Pol) in rhesus macaques (RM)^10^. Vaccines vectored by the RhCMV68-1 strain (RhCMV/SIV) demonstrated impressive vaccine efficacy in wild-type RhCMV-seropositive RM, with 50% of vaccinated animals showing profound early control and eventual clearance of virus from blood and tissues after challenge with pathogenic SIVmac239^10–12^. Of note, the parental RhCMV68-1 strain lacks the RhUL128 and RhUL130 ORFs, resulting in a loss of tropism for epithelial cells which is a change reported as necessary for vaccine immunogenicity^13–16^. Furthermore, analysis of RhCMV/SIV vaccine-induced cellular immunity revealed unconventional major histocompatibility complex (MHC)-II and MHC-E-restricted antiviral CD8 T-cell responses that break previous immunological paradigms^17,18^. More recent reports showed that further attenuation of the RhCMV68-1 vector to limit dissemination of RhCMV-vectored SIV vaccines maintained a high efficacy of 59% and similar T-cell immunogenicity^19,20^. Importantly, antibodies directed to SIV antigens delivered by this vaccine platform have been severely restricted or undetectable in vaccinated RhCMV-seropositive RM^11,12,19^; the mechanistic basis for complete dominance of T-cell over antibody responses remains unknown. These collective findings suggested an opportunity for improvement of efficacy for the RhCMV/SIV vaccine approach by addition of immunomodulators capable of promoting humoral as well as cellular responses.

We hypothesized that activation of innate immunity during vaccination with RhCMV/SIV vaccines would augment vaccine-mediated immunogenicity and protective immunity. To this end, a TLR5 agonist was expressed in the RhCMV68-1 backbone as an in-frame fusion with SIV Gag and evaluated for its ability to increase protective efficacy against SIV challenge. The TLR5 agonist, bacterial flagellin (FliC), exhibits mucosal adjuvant activity for vaccines expressing multiple bacterial and viral antigens by enhancing antibody and, to a lesser degree, cellular responses to vaccine antigens, particularly when expressed as a fusion protein with the immunogen^21–23^. We previously described construction of RhCMV-Gag-FliC, which co-expresses codon-optimized SIVmac239 Gag fused to FliC derived from *Salmonella enterica* serovar Enteritidis and also deleted for the hypervariable domain^24^. These same studies confirmed stable replication of RhCMV-Gag-FliC and TLR5 agonist activity in vitro. Greater inflammation at the site of subcutaneous inoculation distinguished this vaccine when compared to parental RhCMV-Gag and supported adjuvant-associated modulation of innate responses^24^. Studies described herein will compare immunogenicity and efficacy of regimens including RhCMV-Gag versus RhCMV-Gag-FliC when administered to previously RhCMV-seronegative recipients.

## Results

### Robust Gag-specific CD4 T-cell responses in RhCMV-seronegative recipients of a RhCMV/SIV vaccine are diminished by co-expression of a TLR5 ligand

**Figure 1:**
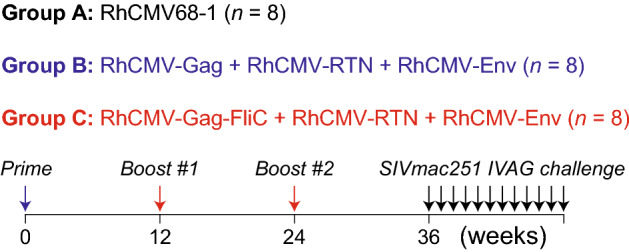
Vaccination protocol. Schematic representation of the study protocol showing RhCMV/SIV or control RhCMV vaccination at 0, 12, and 24 weeks and weekly SIVmac251 challenges (up to 12) starting at 36 weeks after priming immunization.

We compared immunogenicity and protective efficacy of RhCMV68-1-vectored SIV vaccine regimens including RhCMV-Gag^10^ or RhCMV-Gag-FliC^24^ in RhCMV-seronegative RM. Three groups of Specific Pathogen Free level 2 (SPF-2), i.e., RhCMV-seronegative, adult female RM (eight animals per group) were vaccinated with either: empty vaccine vector (RhCMV68.1; Group A), RhCMV68.1 SIV vaccine including RhCMV-Gag, RhCMV-Retanef (RTN), and RhCMV-Env (Group B), or RhCMV-Gag-FliC, RhCMV-Retanef, and RhCMV-Env (Group C) (Fig. 1). Animals positive for MHC-I haplotypes associated with viral-load control^25^ were evenly distributed between groups (Suppl Table S1). Vaccines were administered at weeks 0, 12, and 24. All immunizations were delivered by a combination of subcutaneous (SC; 10^4^–10^5^ PFU) and oral (sublingual and buccal) (PO; 10^5^ PFU) routes with the goal of induction of systemic and mucosal immune responses.

**Figure 2:**
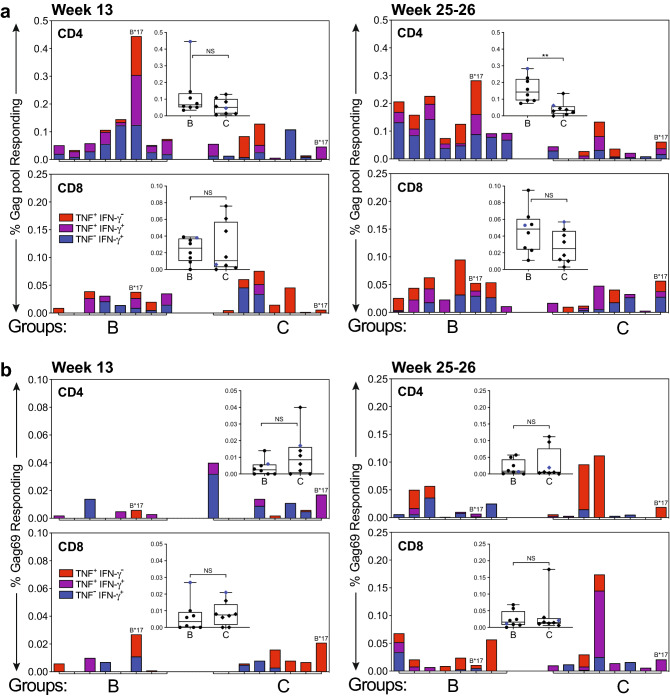
Vaccine induction of antiviral T-cell responses. T-cell responses specific to (**a**) SIV Gag peptide pool and (**b**) Gag69 supertope elicited by RhCVM/SIV vaccines. Responses were evaluated by flow cytometric ICS for TNF and/or IFN-γ expression in gated CD4 or CD8 T-cells after stimulating cryopreserved PBMC thawed the same day, with overlapping peptides for SIV Gag protein or a 15-mer Gag_273-287_(69) peptide. Shown in stacked bar charts are the frequencies of single TNF^+^ (labeled in red), single IFN-γ^+^ (labeled in blue) and TNF^+^IFN-γ^+^ (labeled in purple) responding T-cells. Mamu-B*17-positive RM were labeled in blue in box and whisker plots. n = 8 RM per group. Differences between frequencies of total responding T-cells of groups B and C were analyzed by the nonparametric Mann–Whitney tests (**, *P* < 0.01; NS, not significant).

SIV-Gag-specific T-cell responses were assessed in peripheral blood mononuclear cells (PBMC) using intracellular cytokine staining (ICS) and a pool of 15-mer overlapping peptides spanning SIVmac239 Gag. CD4 and CD8 T-cell responses based on tumor necrosis factor (TNF) and interferon (IFN)-γ expression as demonstrated by a gating strategy and representative scatter plots in Supp Fig. S1. SIV-Gag-specific T-cell responses were comparable between groups B and C at week 13, one week after the first booster immunization (Fig. 2a). Of note, one animal positive for Mamu B*17 in Group B exhibited particularly robust CD4 T-cell responses (Fig. 2a). Based on the modest T-cell responses observed after the first booster immunization, a second boost was administered by both PO and SC routes. Gag-specific CD4 T-cell responses within Group B detected 1–2 weeks after a second booster immunization (Fig. 2a, top right panel) were increased compared to responses to the first booster immunization, and significantly higher in magnitude compared to Group C responses (*P* < 0.01). Gag-specific CD8 T-cell responses were comparable between Groups B and C at this same time point. T-cell responses to Gag in group B were concentrated in the CD4 T-cell compartment at both time points. Despite mucosal delivery of the vaccine, Gag-specific T cell responses were not detected in mononuclear cell (MNC) preparations from colonic biopsies.

Previous reports described unique MHC-II- and MHC-E-restricted Gag-specific CD8 T-cell responses that were elicited by immunization of RhCMV-seropositive RM with the RhCMV/SIV vaccines^18,26^. In this study we assessed responses to a Mamu-E-restricted Gag “supertope” peptide (Gag69) to which all vaccinated RM responded in previous reports^18^. Our results for RhCMV-seronegative RM demonstrated both CD4 and CD8 T-cell responses to the Gag69 supertope peptide, with comparable frequencies in Groups B and C; responses increased after the second booster immunization (Fig. 2b). Gag69-specific response frequencies were of lower magnitude than those shown for the Gag peptide pool for either time point examined (Fig. 2a,b). Interestingly, Gag69-specific responses were comparable between CD4 and CD8 T-cells, although these supertope responses are reportedly restricted by Mamu-E, an MHC-I molecule. Blocking of T-cell responses to Gag69 by loading PBMC with the canonical MHC-E-binding peptide VL9 prior to Gag69 stimulation, was an inconsistent finding in vaccinated RM with no correlation to vaccine protocol, RhCMV serostatus, or other variables. Thus, confirmation of MHC-E restriction of Gag69-specific T-cell responses observed for these vaccine cohorts was not possible.

### Antibody responses to RhCMV68.1-vectored vaccine antigens are determined by wild type RhCMV infection status and the nature of the antigen

**Figure 3:**
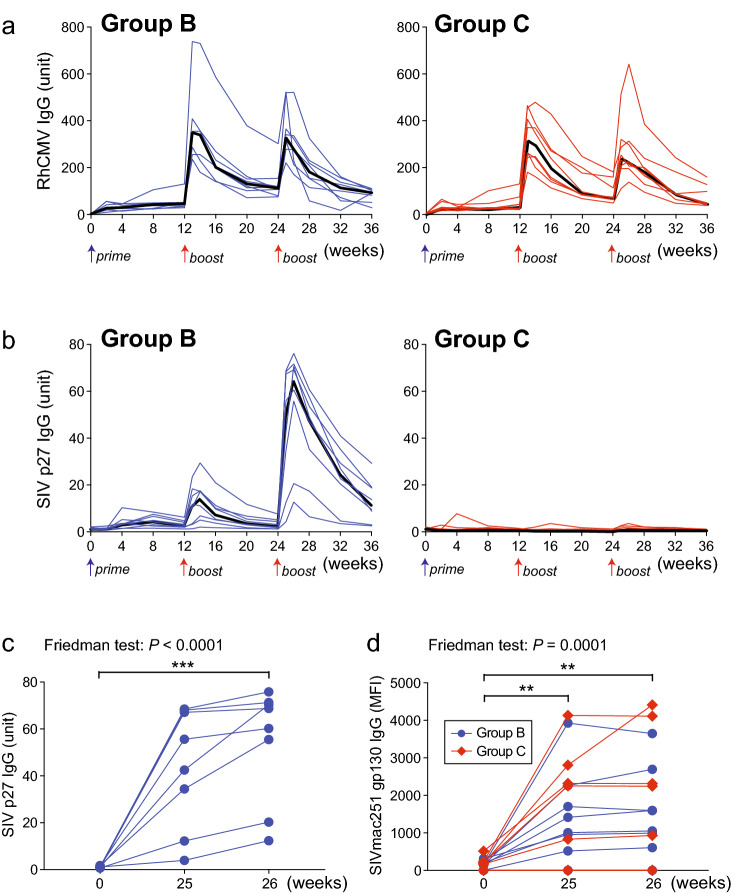
Vaccine induction of antiviral antibodies. Antibody responses elicited by RhCVM/SIV vaccines. Longitudinal binding antibody concentrations specific for (**a**) RhCMV and (**b**) SIV Gag (SIVmac251 p27) in plasma of vaccinated RM. Black lines indicate median values. The second RhCMV SIV vaccine immunization boost significantly enhanced (**c**) anti-SIV p27 antibody concentrations in Group B RM and (**d**) anti-Env (SIVmac251 gp130) antibodies in the majority of Groups B and C animals. Shown are the comparison of IgG concentrations between baseline (week 0) and 1 week (week 25) or 2 weeks (week 26) after the second boost. n = 8 RM per group. The Friedman test was used to determine the significance of matched samples (*P* values shown) and when significant, the Dunn’s multiple comparison test was used to determine the significance of pairwise differences (**, *P* < 0.01; ***, *P* < 0.001).

Previous studies reported very low or absent humoral responses to SIV Env in conventionally raised RhCMV-seropositive RM vaccinated with the RhCMV68.1-vectored SIV vaccine that includes RhCMV-Env^10,12,19^. In contrast, RhCMV-seronegative RM in this study developed detectable antibody responses. Antibody responses to RhCMV vector antigens based on an ELISA^27^ were similar between Groups B and C (Fig. 3a). However, anti-Gag p27 antibody responses as determined by ELISA^28^, were only detected in Group B RM with a progressive increase in titer demonstrated after each immunization. Two RM showed considerably lower antibody concentrations compared to the other six Group B RM (Fig. 3b,c). Induction of antibodies to a RhCMV/SIV vaccine antigen was a novel finding based on published reports, despite use of the same vaccine construct. To test if antibody induction was permitted by absence of previous infection with wild-type RhCMV, we vaccinated additional SPF-2 RhCMV-seronegative RM (*n* = 6) and RhCMV-seropositive RM (*n* = 6) twice with 10^4^ PFU RhCMV-Gag by the SC route. Results of this second study revealed an absence of anti-Gag antibody after two RhCMV-Gag immunizations in RhCMV-seropositive compared to detectable antibody in most RhCMV-seronegative RM (Supp Fig. S2a). Superinfection of RhCMV-seropositive RM was confirmed by detection of de novo Gag-specific T cell responses and boosting of RhCMV-specific responses after a second immunization with RhCVM-Gag (Supp Fig. S2b). Thus, anti-RhCMV vector immunity at the time of immunization prevented humoral responses to the Gag antigen in this vaccine. Finally, anti-Gag antibody responses were marginally or not detected in Group C RM at any time after immunization (Fig. 3b) and were aligned with the significantly weaker Gag-specific T-cell responses observed in group C versus group B RM (Fig. 2a).

Anti-Env (SIVmac251 gp130)-binding antibodies as determined by a custom SIV Env-binding antibody multiplex assay (SIV-BAMA)^29,30^, were detected in the majority of animals in both Groups B and C by 1–2 weeks after the second booster immunization with RhCMV-Env, a component of both vaccine protocols. The magnitude of Env-binding antibody responses was variable within each group but comparable between the two groups (Fig. 3d). One Group B animal did not develop Env-binding antibody. This same RM, along with another RM showing the lowest detectable Env-binding antibody, also demonstrated the lowest anti-Gag antibody concentrations, suggesting that these two animals were poor antibody responders. Additionally, Env-binding antibodies were not detected in two Group C RM. Detection of conclusive antibody responses to both Gag and Env after immunization of RhCMV-seronegative RM with the RhCMV-Gag and RhCMV-Env vaccines demonstrates that pre-existing anti-vector immunity partly determines the intensity of humoral responses to vaccine antigens expressed from RhCMV-vectored vaccines.

### Some RhCMV-seronegative RM vaccinated with the RhCMV68.1-vectored SIV vaccine exhibited superior control of SIV following mucosal challenge

**Figure 4:**
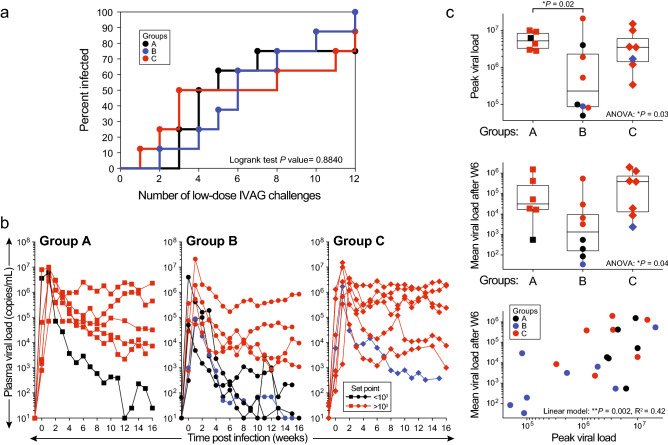
Viral loads after IVAG challenge with SIVmac251. Acquisition of SIV infection and plasma viral load profiles in RhCMV/SIV-vaccinated and control RM. (**a**) Kaplan–Meier curves showing the numbers of challenges required to achieve SIV infection. The onset of infection was defined as positive plasma viral loads (PVL) (10^3^ copies per mL) for two weeks. A nonparametric logrank test indicated that acquisition curves were not significantly different. (**b**) Kinetics of PVL after SIV infection. RM that met controller criteria (PVL set points below 10^3^ copies/mL) were labeled in black and Mamu-B*17-positive SIV-infected RM were labeled in blue. (**c**) Peak and set-point pvls in groups A-C. Analysis represents RM demonstrating detectable viremia; n = 6–8 animals per group. Peak pvls (log values) were significantly lower in Group B RM versus Group A control RM when assessed by ANOVA and post-hoc Dunnett’s tests. Set-point pvls (log of mean values at or after week 6) significantly differed between groups by ANOVA but pairwise comparisons were not significant. Peak and set-point pvls were correlated.

Low dose intravaginal (IVAG) challenge using a previously described SIVmac251 stock and protocol^31^ was conducted 12 weeks after the final immunization (Fig. 1). All Group B RM became viremic, with one animal requiring 12 challenges for detectable infection (Fig. 4a,b). Two control Group A RM that included one positive for Mamu B*17 and one positive for Mamu B*08, and one Group C RM did not demonstrate detectable viremia after 12 IVAG challenges. No significant differences in acquisition rate were noted between groups (Fig. 4a). Among the control Group A RM with detectable viremia, one of six demonstrated significant control of virus load defined by detection of < 10^3^ copies of viral RNA/mL, by eight weeks after detectable infection. Similarly, only one of seven viremic Group C RM showed significant control of virus load by nine weeks post infection. In contrast, four of eight viremic Group B RM showed control of plasma virus by 4–6 weeks post infection with three of the four RM demonstrating virus loads below the limits of detection by 10–15 weeks post infection (Fig. 4b). Peak viral loads were significantly lower among vaccinated Group B RM compared to Group A control RMs (*P* = 0.03) (Fig. 4c), an effect not reported in previous studies using RhCMV-seropositive macaques. Group B RM maintained lower set-point viral loads following presumed immune control of acute viremia (*P* = 0.04). Analysis also revealed a positive correlation between peak and set point viral loads (*P* = 0.002) (Fig. 4c). It should be noted that the single controller in Group C and one of the four controllers in Group B were Mamu-B*17-positive. However, removal of Group B and C Mamu-B*17-positive RMs from the analysis did not significantly impact results for peak and set point virus loads (Supp Fig. S3). In summary, among animals exhibiting detectable viremia in Groups A and C, 50% (4/8) of RM vaccinated with RhCMV68.1-SIV vaccine (group B) showed superior control of plasma virus loads post challenge compared to 17% (1/6) of unvaccinated Group A controls and 14% (1/7) of Group C RM vaccinated with RhCMV-Gag-FliC (Fig. 4b,c).

Hematologic parameters (i.e., CD4/CD8 ratios, CD4 T-cell counts and CD8 T-cell counts) were not significantly different between experimental groups over time (Supp Fig. S4). Ratios of CD4/CD8 frequencies for PBMC, mesenteric lymph node (LN), jejunum and colon collected at time of necropsy, were significantly higher for Group B controllers compared to non-controllers in either Group A, B, or C (Supp Fig. S5). Thus, control of viremia in Group B animals was associated with a sparing effect on CD4 T-cell loss in lymphoid tissues.

### Control of plasma viremia was associated with waning anti-Gag antibody in vaccinated RM

**Figure 5:**
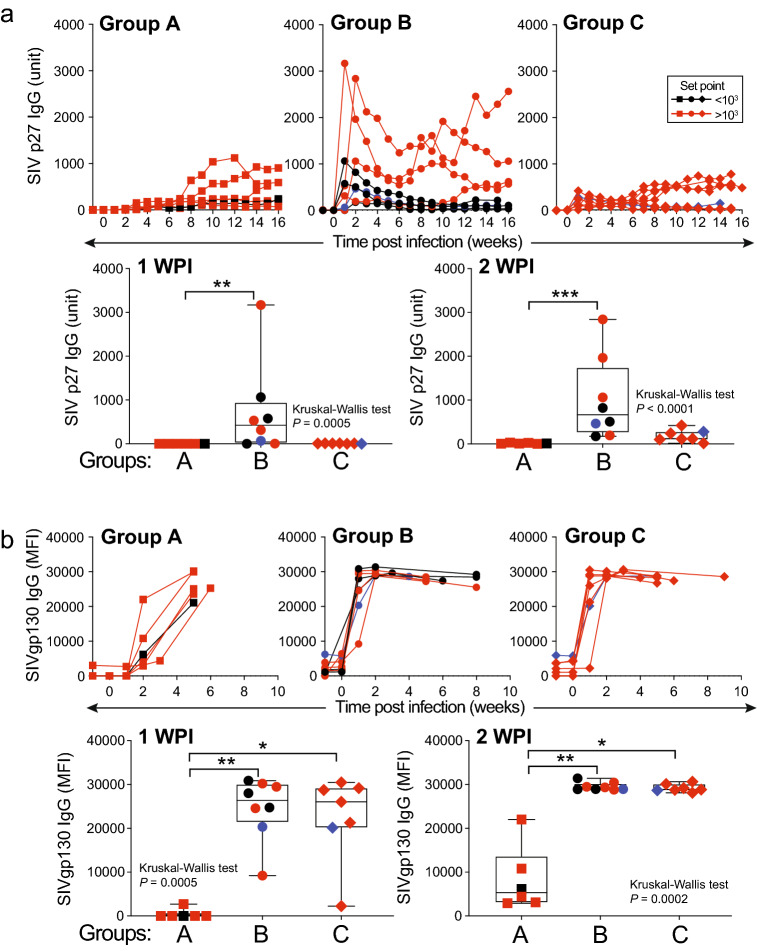
Anti-viral antibody responses after IVAG challenge with SIVmac251. Kinetics of antibody responses to (**a**) Gag (p27) and (**b**) Env (gp130) following SIV infection in control and RhCMV/SIV-vaccinated RM. RM that met controller criteria were labeled in black and Mamu-B*17-positive RM were labeled in blue. Analysis represents RM demonstrating detectable viremia; n = 6–8 animals per group. Comparative analyses of anti-p27 antibody concentrations and anti-gp130-binding activity between all groups at 1 and 2 WPI were performed by Kruskal–Wallis test (*P* values shown) followed by Dunn’s multiple comparison test for pairwise comparison (*, *P* < 0.05; **, *P* < 0.01; ***, *P* < 0.001).

Strong anamnestic antibody responses to Gag were observed in group B RM at one and two weeks after infection, whereas increases in anti-Gag antibody for this same time point were considerably weaker for group C and nearly absent in group A animals (Fig. 5a). This finding was not unexpected given that anti-Gag antibody responses were undetectable in group C animals after immunization. In contrast, anamnestic anti-Env-binding antibody responses emerged within one to two weeks post infection and were significantly greater than anti-Env responses for group A animals at one and two weeks after infection (Fig. 5b). However, anamnestic anti-Env antibodies were comparable between groups B and C and between virus controllers and non-controllers in both groups. Vaccine-induced anti-Env antibody concentrations before challenge did not correlate with plasma viral loads after challenge as the two low responders in Group B included both a controller and non-controller RM. Anti-Env antibody was not detected in unvaccinated Group A controls until two to three weeks post infection (Fig. 5b).

Interestingly, by two weeks after detectable infection, anti-Gag antibody responses in Group B controllers progressively declined in magnitude over time compared to non-controllers within the same group (Fig. 5a). Decline of anti-Gag antibody responses over time in Group B virus controllers was contemporaneous with decreasing virus loads in these same animals. However, anti-Gag antibody responses did not distinguish controllers from non-controllers in Groups A and C (Fig. 5a).

### SIV-specific CD8 T-cell responses in vaccinated RM did not correlate with protection

**Figure 6:**
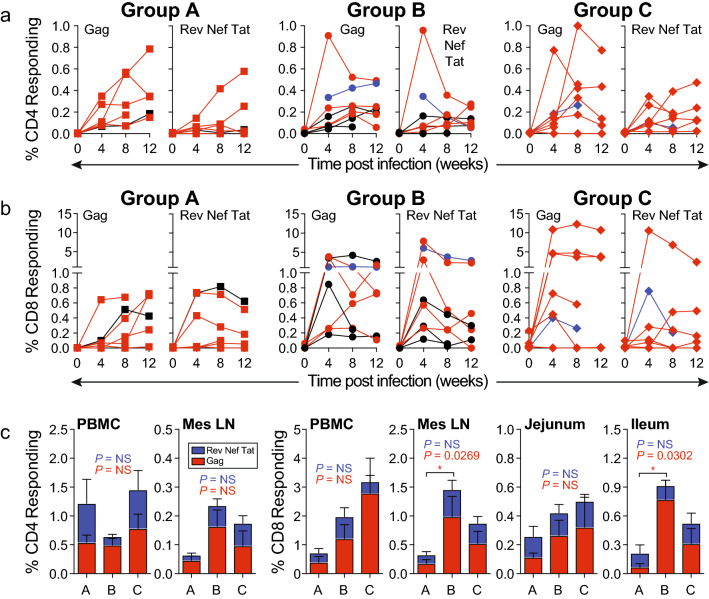
Antiviral T-cell responses after IVAG challenge with SIVmac251. Cellular immune responses following SIV infection. Kinetics of SIV-Gag- and SIV-RTN-specific (**a**) CD4 and (**b**) CD8 T-cell responses in freshly isolated PBMC after SIV infection. Responses were determined by ICS for both TNF and IFN-γ expression after stimulating with overlapping peptide pools for each SIV protein. RM that met controller criteria were labeled in black and Mamu-B*17-positive RM were labeled in blue. Frequencies represent T cells stining positive for both TNF and IFN-γ expression. (**c**) Comparison of the SIV-specific CD4 and CD8 T-cell populations of PBMC and MNC freshly isolated from indicated tissues assessed at necropsy of SIV-infected RM. Time points for necropsy varied from 17–41 weeks (median 28 weeks) after SIV infection. Analysis represents RM that demonstrated detectable viremia; n = 6–8 animals per group. Shown in stacked bar charts are the mean frequencies (+ SEM) of responses to SIV-Gag (red) and SIV-RTN (blue). The Kruskal–Wallis test was used to determine the significance of overall differences of Gag- and RTN-specific T-cell responses, with P values shown in red and blue, respectively. If significant, the Dunn’s post test was used to determine the significance of pairwise differences (*, *P* < 0.05; NS, not significant).

Circulating CD4 and CD8 Gag-specific T-cell responses observed after immunization and before challenge did not correlate with control of viremia after challenge. Although Gag-specific CD4 T-cell responses for group B controllers after the second boost were significantly greater compared to Group C non-controllers, Group B controller responses were not different when compared to Group B non-controllers (Supp Fig. S6). Furthermore, circulating CD4 and CD8 T-cell responses were not significantly different between vaccine groups for either CD4 or CD8 T-cells during either acute or early set point phases of infection after challenge (Fig. 6a,b). Of note, both CD4 and CD8 T cell responses for Gag and RTN after challenge and one week prior to detectable plasma viremia (time zero of infection) (Fig. 6a,b) had significantly declined compared to responses demonstrated one to 2 weeks after the second immunization boost, particularly for Group B CD4 T cell responses (Fig. 2a). Similarly, combined Gag- and RTN-specific CD4 T-cell responses in blood and MNC isolated from mesenteric LN were not statistically different between groups at time of necropsy. In contrast, blood and tissue CD8 T-cell responses at necropsy tended to be greater for Groups B and C, reaching significance for CD8 T-cell responses in mesenteric LN and ileum in Group B RM when compared to control Group A (Fig. 6C). Importantly, these enhanced SIV-specific CD8 T-cell responses observed for vaccinated group B RM did not distinguish vaccinated controllers from non-controllers. Regardless, these findings did suggest that immunization successfully primed CD8 T-cell responses in mesenteric LN and gut mucosa.

## Discussion

Remarkable rates of 50–59% efficacy have been reported for the RhCMV68-1-vectored SIV vaccine and an attenuated version vectored by ΔRH110 RhCMV. This success was obtained despite restricted or absent antibody responses to vaccine antigens^10–12,19^. Our current study found that four of eight viremic RM (50%) vaccinated with the RhCMV68-1-vectored SIV vaccine demonstrated superior control of virus loads after challenge, as defined by a significant decline in plasma viral loads (Fig. 4b), a parallel decline in anti-Gag antibody responses (Fig. 5a), and sparing of CD4 T cells in blood and tissues (Supp Fig. S5) at later time points. Furthermore, mean peak and set point viral loads were lower for Group B RM vaccinated with this RhCMV/SIV vaccine (Fig. 4c). These findings represent the first report of an efficacy trial with the RhCMV68-1-vectored SIV vaccine in RhCMV-seronegative RM. Therefore, vaccination occurred in the absence of pre-existing vector immunity and modulation of host immune responses by chronic RhCMV infection. It is noteworthy that in this study, up to 14 weeks were required for virus loads to fall below 10^3^ copies per ml of plasma in RM controlling virus loads after challenge (Fig. 4b). These results differ significantly from previous studies reporting complete control of viremia within 1–2 weeks of peak viremia among previously RhCMV-seropositive RM immunized with RhCMV/SIV vaccines^10–12,19^. Also, previous studies confirmed clearance of pathogenic SIVmac239 challenge virus from all tissues analyzed by 28 to 40 weeks post-infection^12,19^. RM in the current study were not maintained for such an extended period or tested for viral clearance from tissues. Factors that may account for these differences in virus-control kinetics include the significantly shorter time frame of 12 weeks between last immunization and first challenge for this current trial, compared to 45–88 weeks reported for previous trials^10–12,19^; immunization of RhCMV-seronegative RM in contrast to RhCMV-seropositive animals; and use of a SIVmac251 challenge versus a SIVmac239 challenge utilized in previous studies. Other possible factors contributing to differences in virus control kinetics could relate to the nature and magnitude of Gag-specific T cell responses observed in vaccinated RM in this current trial compared to previous reports of RhCMV/SIV vaccines. Certain differences in the ICS assay, such as testing of cryopreserved PBMC and stimulation with only Gag and Retanef peptides, preclude direct comparison of our findings for antiviral T cell response with those of previous reports for this vaccine approach^10–12^. However, SIV-specific T cell responses measured after challenge and immediately prior to detectable SIV infection were severely restricted (Fig. 6a,b) compared to those detected earlier at one to two weeks after the second booster immunization (Fig. 2a). These highly restricted antiviral T cell responses observed 12 weeks after the final immunization and during challenge contrasted with sustained vaccine-induced T cell responses over months to years reported in previous studies^11,12,19^. This observation possibly suggested either a diminished durability of vaccine-induced T cell responses in RhCMV-seronegative RM, or the need of additional time between immunization and challenge for establishment of a resilient vaccine virus infection and vaccine-induced T cell responses. It is also important to note that previous reports^10–12,19^ have not confirmed anti-viral T cell responses as a correlate of vaccine efficacy. Regardless, multiple variables including pre-immunization RhCMV vector immunity and time frame allowed for maturation of vaccine-induced immunity may warrant further examination.

An unexpected but significant finding for this vaccine trial involving RhCMV-seronegative RM was detection of anti-Gag antibodies in Group B animals, as well as anti-Env antibodies in Groups B and C (Fig. 3b,c). Observations of antibody responses after vaccination were further supported by detection of anamnestic antibody responses directed to Gag in Group B and Env in Groups B and C at early time points after infection post challenge (Fig. 5a,b). These results contrasted with findings of previous RhCMV/SIV vaccine trials that utilized predominantly RhCMV-seropositive RM^10–12,19^, and our separate immunogenicity study with RhCMV-seropositive RM (Supp Fig. S2a). These noteworthy findings demonstrate an effect of anti-vector immunity and/or chronic RhCMV infection on humoral responses to vaccine antigens delivered by RhCMV vectors. We recently reported that chronic subclinical viral infections including RhCMV impacted blood immune profiles, gut microbiota and humoral immune responses to an influenza vaccine in RM^32^. Additionally, a recent report revealed that prior immunity to mouse CMV (MCMV) conferred a negative impact on vaccine-associated humoral immune responses and efficacy of a MCMV-vectored vaccine expressing a Friend virus (FV) Env immunogen (MCMV.env) in mice highly susceptible to FV^33^. Furthermore, these studies also reported that a longer time frame between vaccination and challenge was required for MCMV.env vaccine efficacy. It is important to note that although our studies also revealed an impact on vaccine-induced humoral responses conferred by vector immunity, anti-ENV-binding antibody responses post immunization were of low magnitude and anti-ENV antibody responses post challenge did not correlate with virus control. Because humoral immunity is considered a necessary component of a successful HIV vaccine, elucidation of mechanisms by which pre-existing immunity and chronic CMV infection impact such responses elicited by vaccines based on RhCMV or human CMV (HCMV) vectors is needed.

In an effort to improve vaccine immunogenicity and efficacy, RM were immunized with a RhCMV68-1-vectored SIV vaccine that replaced RhCMV-Gag with RhCMV-Gag-FliC. This strategy was based on the hypothesis that co-expression of a TLR5 ligand would enhance vaccine-induced humoral and cellular responses through activation of innate responses associated with TLR5 stimulation^21–23^. However, this strategy failed to achieve enhanced immunogenicity in either systemic or mucosal tissues, or to induce protection against pathogenic SIVmac251. These outcomes were unexpected but yielded important insights regarding modification of CMV-based vectors. First, anti-Gag antibody responses were not detected in RM immunized with RhCMV-Gag-FliC but were detected in RM immunized with the RhCMV-Gag vaccine (Fig. 3b). Possible explanations for this outcome include reduced in vivo expression of the Gag-FliC antigen due to virus instability, reduced virus replication, or both. Second, TLR5 agonists activate multiple cell types, eliciting a wide range of innate cytokines and triggering the NLRC4/NAIP5-dependent inflammasome pathway^21,34^. In support of these TLR5 agonist activities, histopathologic analysis of skin biopsies harvested at the site of inoculation revealed a proinflammatory response of higher magnitude elicited by RhCMV-Gag-FliC compared to RhCMV-Gag, as described in our previous report that included samples from the current study^24^. Thus, an exuberant proinflammatory response associated with the FliC-adjuvanted vaccine may have altered the local immune environment, resulting in partial clearance of vaccine virus and/or modified presentation of vaccine-expressed antigens. Furthermore, RhCMV and HCMV encode multiple gene products with immune-modulating functions including down-regulation of MHC-I molecules, evasion of natural killer cells, and viral analogs of cytokines and chemokines. These virus-encoded activities are thought to play a critical role in establishment and persistence of CMV infections, as well as the phenomenon of CMV superinfection^35–37^. Possibly, antagonism between vector-associated immune evasion and TLR5 agonist activities may have led to reduced vaccine replication and immunogenicity.

Although Gag-FliC expression from RhCMV-Gag-FliC was sufficient to elicit detectable Gag-specific T-cell responses, these responses were not boosted after a second booster immunization and were lower than those elicited by RhCMV-Gag at this same time point (Fig. 2a). Taken together, these findings suggest a significant reduction in vaccine expression of Gag-FliC immunogen after both priming and boosting immunizations, which may have contributed to the lower efficacy of the RhCMV-Gag-FliC-based vaccine. Overall, these results suggest that adjuvant-associated modulation of innate responses to RhCMV-vectored vaccines may unpredictably impact immunogenicity and efficacy. These findings also confirmed the importance of the RhCMV-Gag component for efficacy associated with the RhCMV/SIV vaccine strategy.

Another unexpected finding was the nature of vaccine-induced T-cell responses directed to the MHC-E-restricted Gag69 supertope. Although detected, responses were inconsistent and of lower magnitude (Fig. 2b) than expected based on previous reports with this vaccine^17,19,20,26^. Furthermore, both CD4 and CD8 T-cell responses were detected for this supertope and MHC-E restriction could not be confirmed for RM within this study due to inconsistent blocking activity by the MHC-E-binding peptide, VL9. A rigorous examination of MHC-E-restricted T-cell responses was beyond the scope of this study and would be necessary to draw definitive conclusions. Nevertheless, these findings raised important questions regarding factors that influence induction of these recently described novel responses. Variables that might contribute to the different responses to the Gag69 supertope in this study include the use of RhCMV-seronegative RM, assay of cryopreserved PBMC, or other variables yet to be determined.

Immune correlates of viremia control were not apparent from analysis of SIV-specific T-cell responses or anti-Env antibody responses, either post vaccination (Supp Fig. S6a,b) or post challenge (Fig. 6a–c). SIV-specific CD8 T-cell responses in blood and mesenteric LN harvested at terminal time points did distinguish Group B RM from the control Group A, but were not significantly different from responses detected for Group C (Fig. 6c). Definitive immune correlates of efficacy associated with the RhCMV SIV vaccine were also not determined in previous reports^10–12,19^, where both vaccinated controllers capable of SIV clearance and vaccinated non-controllers demonstrated MHC-E-restricted CD8 T cell responses. Conclusive immune correlates for this vaccine approach remain to be elucidated and may relate to innate as well as adaptive responses.

This study reached the following significant conclusions. First, immunization of RhCMV-seronegative RM with a RhCMV68-1-vectored SIV vaccine resulted in exceptional control of plasma viremia after IVAG challenge with SIVmac251 in 50% of RM, lending additional support to this platform for AIDS vaccine development. However, of equal significance, our investigation revealed an efficacy that was qualitatively different with vaccinated macaques demonstrating a control of challenge virus with slower kinetics compared to the immediate control described in previous reports^10–12,19^. Second, pro-inflammatory adjuvants such as a TLR5 ligand co-expressed with a vaccine antigen delivered by a RhCMV vector can result in unpredictable immunogenicity and efficacy, which may result from adjuvant-mediated interference with vector replication or immunomodulatory properties. Third, RhCMV-vectored vaccine induction of humoral responses in RhCMV-naive hosts contrasts with findings in RhCMV-infected hosts and demonstrates that pre-existing vector immunity significantly impacts CMV-vectored vaccine immunogenicity. Fourth, the time required for establishment of fully protective RhCMV/SIV vaccine-induced immunity may be a factor in vaccine efficacy and remains to be determined. In summary, these findings are novel and impactful, address a promising HIV vaccine candidate, and underscore the need for further investigation of host/vector interactions that impact immunogenicity and efficacy of CMV-vectored vaccines.

## Methods

### Research animals

The vaccine trial utilized 24 outbred female SPF-2 RM (Macaca mulatta) (23 Indian-origin and 1 Indian- and Chinese-origin mix) ranging from 3 to 7 years of age. MHC class I genotyping for Mamu was alleles was performed by the AIDS Vaccine Research Laboratory, University of Miami, Miller School of Medicine. SPF-2 RM were maintained as free of infection with RhCMV, simian foamy virus (SFV), herpes B virus (BV), type D simian retrovirus (SRV), simian T-lymphotropic virus (STLV), and SIV. A second immunogenicity trial for the RhCMV-Gag vaccine utilized one group of SPF-2 RM (male and female; n = 6; 1–2 year of age) and a second age-matched group of six conventionally raised non-SPF RhCMV-seropositive RM (male and female; n = 6).

### RhCMV/SIV vaccines

The construction and characterization of the RhCMV/SIV vaccines expressing SIVmac239 Gag, RTN, and Env were previously described^10^. Construction and characterization of the RhCMV68-1 vector expressing a SIVmac239-Gag and Salmonella enterica serovar Enteritidis FliC (flagellin) fusion protein (RhCMV-Gag-FliC) were also previously reported^24^. All virus stocks were titered by our standard plaque reduction assay using telomerized-rhesus fibroblasts^38^, that differs from titering methods used by Hansen et al.^10^.

### Vaccinations

For the vaccine trial, animals were primed with either RhCMV68-1 (control Group A) or RhCMV/ SIV vaccines subcutaneously (10^4^ PFU per vaccine) and orally (10^5^ PFU per vaccine) and boosted subcutaneously and orally (10^5^ PFU per vector for both routes) at weeks 12 and 24. For immunogenicity studies in SPF-2 (n = 6) or non-SF (n = 6) RM, RhCMV-Gag was delivered subcutaneously at a dose of 10^4^ PFU at weeks 0 and 16.

### Blood and tissue collection and processing

EDTA-treated venous blood collected from all animals at various time points were processed for plasma and peripheral blood mononuclear cells (PBMC) isolation by Accu Paque gradient centrifugation (Accurate Chemical & Scientific Corp). PBMC were cryopreserved by resuspension freezing media containing 10% dimethly sulfoxide (DMSO) in fetal calf serum for assay of T cell response assays. Mononuclear cells (MNC) from lymph nodes (LN) or intestinal mucosa were isolated after incubation in digesting solution containing 50 U/mL DNase I and 250 U/mL type I collagenase (Worthington Biochemical Corp) or 0.5 mg/mL type II collagenase (Sigma-Aldrich) for 30 min and mechanically disrupted in gentleMACS C tubes (Miltenyi Biotec) and 70 μm cell strainers (Thermo Fisher Scientific).

### Antibody assays

The binding IgG titers for RhCMV antigens and SIV Gag p27 were quantified by in-house ELISAs as previously described^28^. Plasma IgG binding to SIVmac251 gp130 and gp70 V1V2, SIVmac239 gp120 and gp70 V1V2, SIVsmE660 gp140 and gp70 V1V2 were determined by a custom SIV binding antibody multiplex assay (SIV_BAMA) as previously described^29,30^.

### ICS assays

SIV-specific CD4 and CD8 T-cell responses were measured in PBMC and tissue MNC by flow cytometric intracellular cytokine analysis. Sequential 15-mer peptides (overlapping by 11 amino acids) comprising the SIVmac239 Gag (kindly provided by Drs. Scott Hansen and Louis Picker from VGTI, OHSU) and Rev, Tat, and Nef (provided by NIH AIDS Reagent Program) proteins or a 15-mer Gag_273-287_(Gag69) peptide (INTAVIS Bioanalytical Instruments AG) were used in the presence of co-stimulatory CD28 and CD49d monoclonal antibodies (clone L293 and L25; BD Bioscience). Cells were incubated with peptides and co-stimulatory antibodies alone for 1 h, followed by adding Brefeldin A (Sigma-Aldrich) for an additional 8 h. Cells incubated with co-stimulatory antibodies in media with DMSO but without peptides served as a control for background subtraction. Whole blood or cells from T-cell assays were stained with fluorochrome-conjugated monoclonal antibodies against human (rhesus macaque cross-reactive; purchased from BD Bioscience) cell markers including CD3 (SP34-2), CD4 (L200), CD8 (SK1), CD20 (L27), CD14 (M5E2), CD16 (3G8), TNF (Mab11), and IFN-γ (B27). Lysis and fixation of whole blood samples were performed by the TQ-Prep Workstation and IMMUNOPREP reagent system (Beckman Coulter). Data were analyzed and illustrated using FlowJo software (BD Bioscience). Cells were acquired using a LSRFortessa cell sorter operated by FACSDiva software (BD Bioscience) with a minimum acquisition of 10^5^ live T cells. Data were analyzed using FlowJo software (BD Bioscience). Frequencies of responding cells found to be less than 0.01% were considered very low and equivocal.

### SIV mac251 challenge

All RM in the vaccine trial were challenged IVAG with SIVmac251 at 12 weeks after the last boost using a repeated (weekly) low dose challenge protocol. In brief, 1 mL of 1:20 diluted pathogenic SIVmac251 stock (provided by Dr. Nancy Miller from NIAID, Desrosiers stock-batch number 2010-Day 8, Lot # 305342b), equivalent to a titer of 5 × 10^3^ TCID_50_ in rhesus 221 cells, was delivered IVAG to each RM every week until infected or up to 12 challenges. Infection was defined by detection of plasma virus load of 10^3^ SIV RNA copies per mL. Plasma SIV viral loads were measured weekly, with challenge continued for one week after detection positive of SIV RNA. Plasma SIV RNA levels were measured by a *gag*-targeted quantitative real-time/digital RT-PCR, designed to prevent potential cross reactivity between RhCMV-Gag and SIV, as previously described^39–41^. A total of 6 replicate reactions were analyzed per extracted RNA sample for assay thresholds of 15 SIV RNA copies in 1 mL plasma.

### Statistics

Statistical and graphic analysis and graphing were conducted with GraphPad Prism 8 software (GraphPad Software, Inc) or in the R programming language (R: A language and environment for statistical computing. https://www.R-project.org/). Comparisons of matched samples were performed with Friedman nonparametric tests followed by Dunn’s multiple comparison tests. Protection against acquisition of SIVmac251 infection was analyzed by logrank tests. Overall comparisons between the control and vaccine groups were performed with ANOVA parametric or Kruskal–Wallis nonparametric tests. If the differences were significant, Dunnet’s (for ANOVA) or Dunn’s (for Kruskal–Wallis) post tests were conducted to determine the significance of pairwise differences between groups. For all tests, P < 0.05 was considered significant.

### Study approval

All studies were conducted at the California National Primate Research Center (CNPRC) and were approved in advance by the University of California Davis (UC Davis) Institutional Animal Care and Use Committee (IACUC). All experiments were performed in accordance with UC Davis relevant guidelines and regulations including approved Biological Use Authorizations required by the UC Davis Safety Services for handling of nonhuman primate tissues, SIV virus stocks, and RhCMV vaccine vectors.

## Supplementary information

Supplementary file1

## Data Availability

All data needed to evaluate the conclusions in the paper are present in the paper and/or the Supplementary Materials. Additional data are available from the corresponding author on reasonable request.
