# Supplementary material for: RhCMV serostatus and vaccine adjuvant impact immunogenicity of RhCMV/SIV vaccines
Source: Sci Rep. 2020 Aug 20;10:14056. doi: 10.1038/s41598-020-71075-x (PMC7441386; doi:10.1038/s41598-020-71075-x)
Supplement: Supplementary file 1 — Supplementary file1 [file 41598_2020_71075_MOESM1_ESM.pdf]

Supporting Information

REVISION Manuscript beac5368-8250-458f-a9bc-50b1d35cf864

RhCMV serostatus and vaccine adjuvant impact immunogenicity of RhCMV/SIV vaccines

W. L. William Chang[1,2,3], Jesse D. Deere[3], Hung T. Kieu[1,2,3,4], Luis D. Castillo[1,2,5], Kawthar Machmach[2,3,6], Xiaoying Shen[7], Georgia D. Tomaras[7], Barbara L. Shacklett[3,8], Peter A. Barry[1,2], Dennis J. Hartigan-O'Connor[2,3*], Ellen E. Sparger[4**]

Supplementary Table S1. MHC class I genotype of RM in each group

Group	A* 01	A* 02	A* 08	A* 11	B* 01	B* 03	B* 04	B* 08	B* 17	B* 29
A	-	-	-	-	-	-	-	-	-	-
A	-	-	-	-	+	-	-	-	+	-
A	+	-	-	-	-	-	-	-	-	-
A	-	-	+	+	+	-	-	+	-	-
A	-	-	-	-	+	-	-	-	-	-
A	+	-	-	-	-	-	-	-	-	-
A	-	-	-	-	+	-	-	-	-	-
A	-	-	-	-	+	-	-	-	-	-
B	-	-	-	-	-	-	-	-	-	-
B	+	-	-	-	-	-	-	-	-	-
B	-	-	-	-	-	-	-	-	-	-
B	-	-	+	-	-	-	-	-	-	-
B	+	-	-	-	+	-	-	-	-	-
B	+	-	-	+	-	-	-	-	+	+
B	-	-	-	-	+	-	-	-	-	-
B	-	-	+	+	-	-	-	-	-	-
C	+	-	-	-	+	-	-	-	-	-
C	-	-	-	-	+	-	-	-	-	-
C	+	-	-	-	-	-	-	-	-	-
C	-	-	-	-	-	-	-	-	-	-
C	-	-	+	-	+	-	-	-	-	-
C	-	-	-	-	-	-	-	-	-	-
C	-	-	+	-	+	-	-	-	-	-
C	-	-	-	-	+	-	-	-	+	+

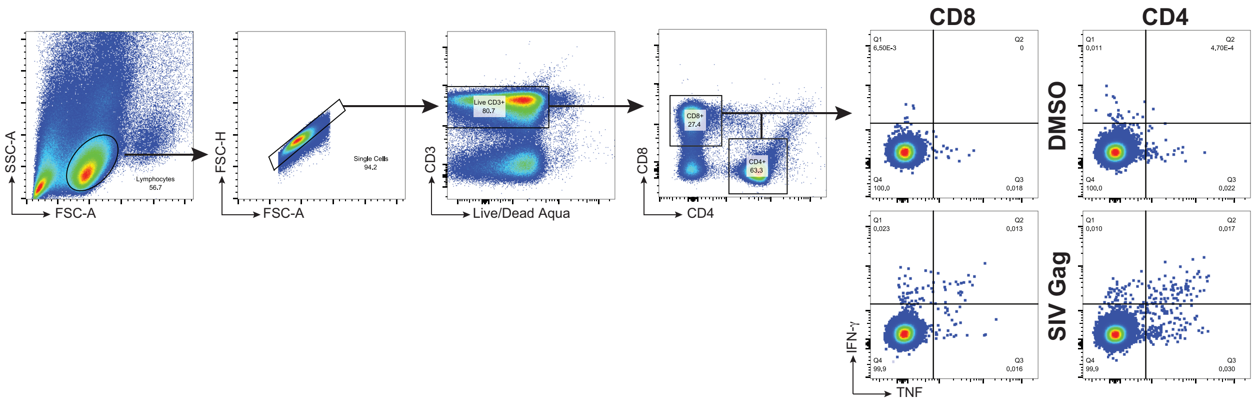

Supplementary Figure S1. Flow cytometric ICS plots showing SIV Gag-specific T-cell responses. Representative flow cytometric ICS plots demonstrating CD8 and CD4 T-cell responses to SIV Gag peptides are shown for one SIV-infected RM. The frequencies of IFN-γ and/or TNF-positive cells were assessed after gating on live, $CD3^+$, $CD4^+$ or $CD8^+$ lymphocytes. Cells incubated with co-stimulatory antibodies and DMSO without peptides served as a control for background subtraction.

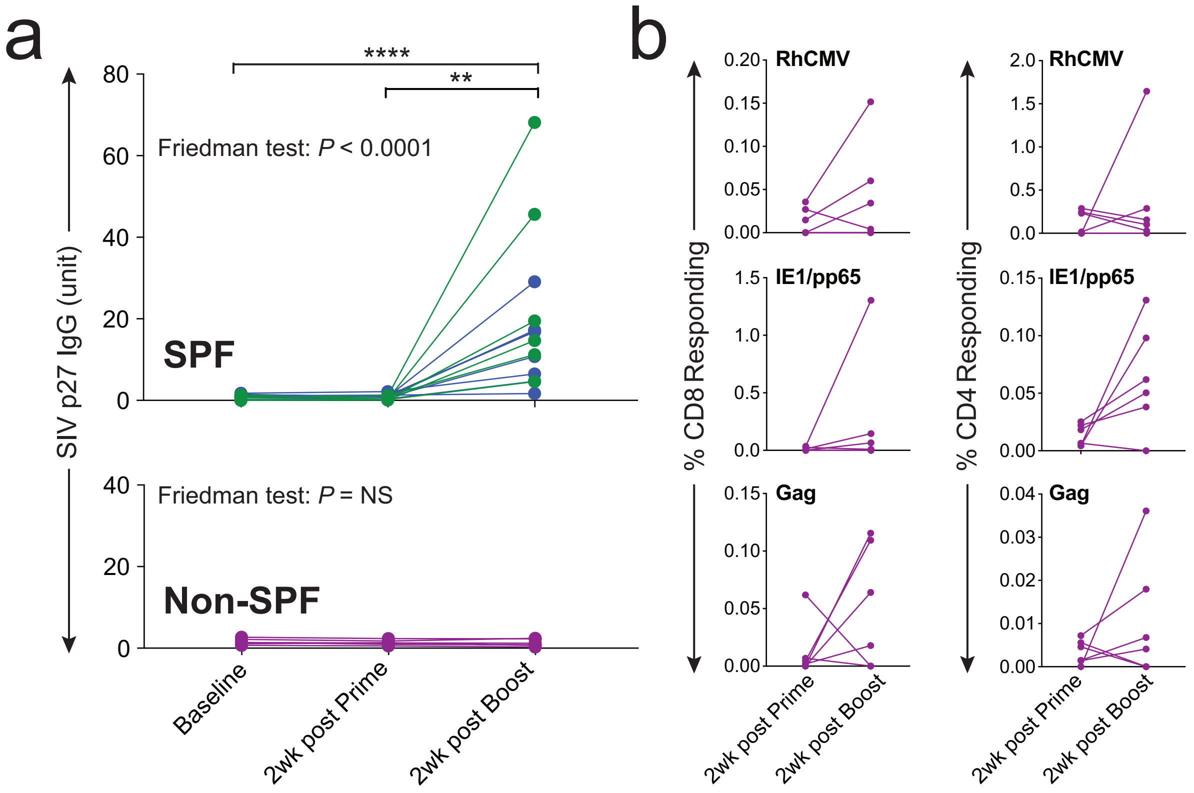

Supplementary Figure S2. Induction of anti-SIV Gag antibodies in RhCMV-seronegative RM by vaccination with RhCMV-Gag. 14 SPF RM (including 8 RM in Group B labeled in blue and 6 additional RM labeled in green) and 6 non-SPF (RhCMV-positive) RM (labeled in purple) received an RhCMV-Gag vaccine priming immunization at week 0 and boost at week 12 (n = 14) or 16 (n = 6). (a) Anti-SIV Gag (p27) IgG concentrations in plasma samples were assessed by ELISA. The Friedman test was used to determine the significance of matched groups (P values shown; NS, not significant) and when significant, the Dunn's multiple comparison test was used to determine the significance of pairwise differences (**, $P < 0.01$; ****, $P < 0.0001$). (b) T-cell responses in PBMC of 6 non-SPF RM at two weeks after RhCMV-Gag prime and boost. Responses were determined by flow cytometric ICS for both TNF and IFN-γ expression after stimulating with heat-inactivated RhCMV virions or overlapping peptide pools for RhCMV IE1 and pp65 proteins or SIV Gag.

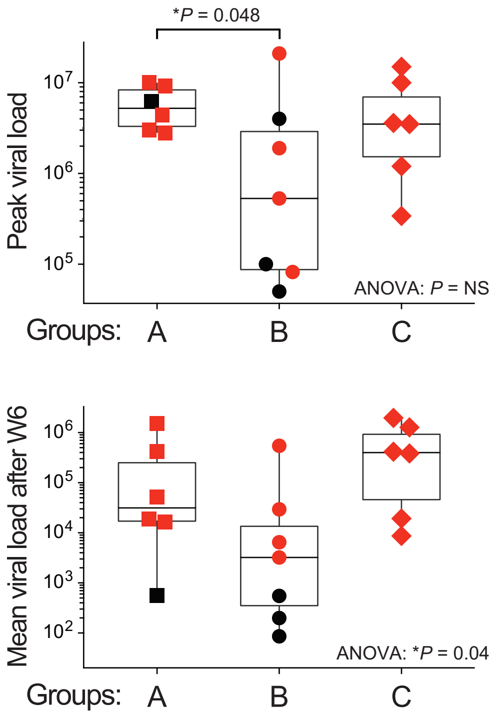

Supplementary Figure S3. Comparison of plasma viral load (PVL) of animals in Groups A-C when excluding two *Mamu-B*17*-positive macaques. Analyses include the peak PVL and mean PVL at or after week 6. Analysis represents RM demonstrating detectable viremia; n = 6-8 animals per group. A single, uncorrected *t* test indicates significantly lower peak PVL in Group B vs. control animals (Group A); however, analysis of variance (ANOVA) indicates no significant difference when all are considered. Set-point PVL significantly differed between groups by ANOVA but pairwise comparisons were not significant.

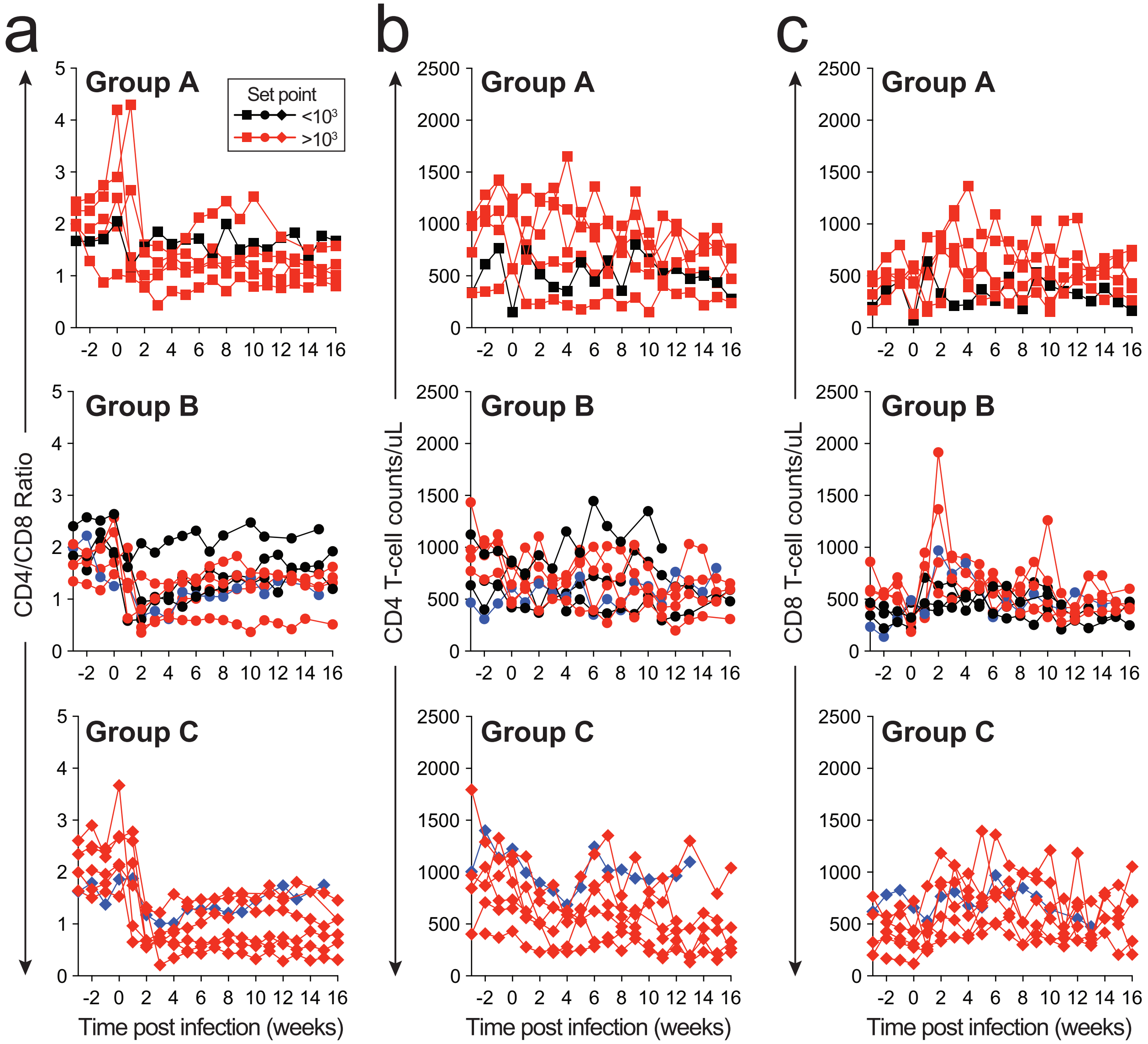

Supplementary Figure S4. Dynamics of circulating T-cell changes during the course of SIV infection in control and RhCMV SIV-vaccinated RM. Shown are CD4/CD8 T-cell ratios (a), absolute counts of CD4 T cells (b) and CD8 T cells (c) in control (Group A) and vaccinated (Groups B and C) RM. Analysis represents RM demonstrating detectable viremia; n = 6-8 animals per group. RM in each group that met controller criteria (PVL set points below 10^3 copies/mL) were labeled in black and the *Mamu-B*17*-positive SIV-infected RM were labeled in blue.

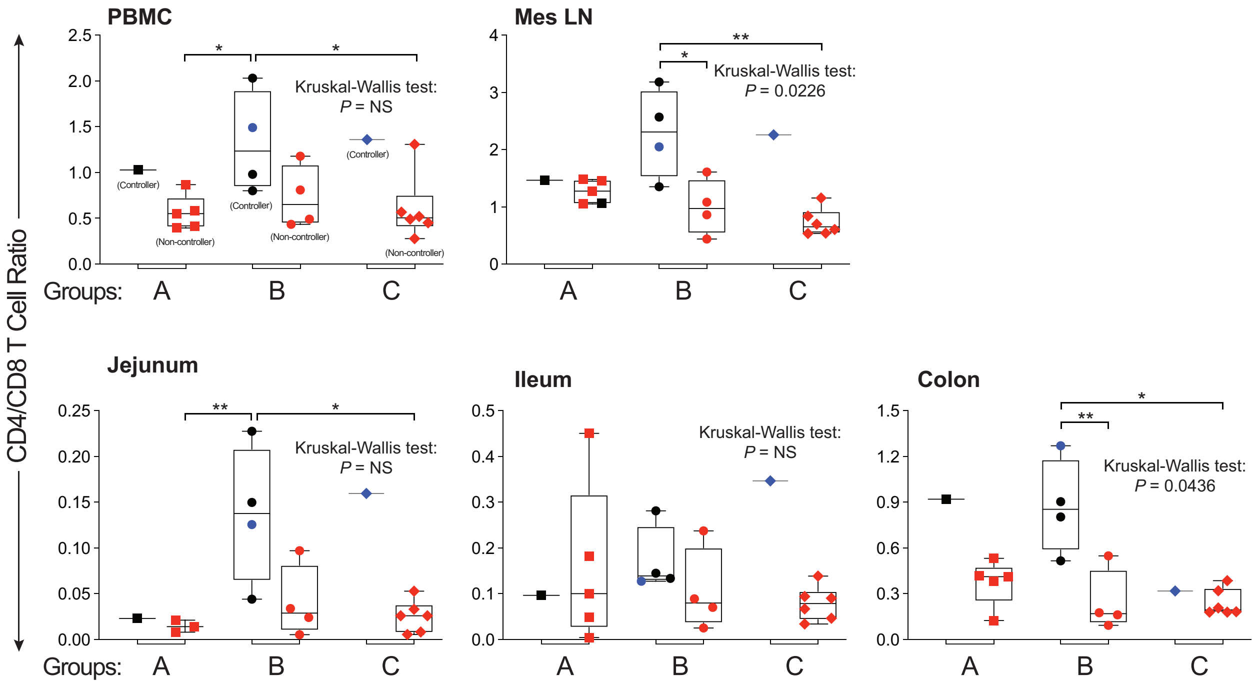

Supplementary Figure S5. Comparative analysis of CD4 and CD8 T-cell ratios in blood and lymphoid tissues of SIV-infected RM at necropsy. CD4 and CD8 T-cell ratios in PBMC and mesenteric and gut lymph nodes measured at necropsy are shown for control (Group A) and vaccinated (Groups B and C) RM. The frequencies of CD4 and CD8 T-cells within the gated $CD3^+$ lymphocyte population were determined by flow cytometric analyses. RM that met controller criteria were labeled in black (two *Mamu-B*17*-positive RM labeled in blue) and the non-controllers were labeled in red. Overall differences were determined by Kruskal-Wallis test (*P* values shown; NS, not significant) and the uncorrected Dunn's test was used to determine the significance of pairwise differences (*, $P < 0.05$; **, $P < 0.01$). Time point of necropsy after SIV infection (weeks): Group A controller (18); Group A non-controllers (17, 17, 18, 18, 36); Group B controllers (28, 33, 37, 40); Group B non-controllers (33, 33, 39, 41); Group C controller (28); Group C non-controllers (16, 20, 21, 21, 22, 28).

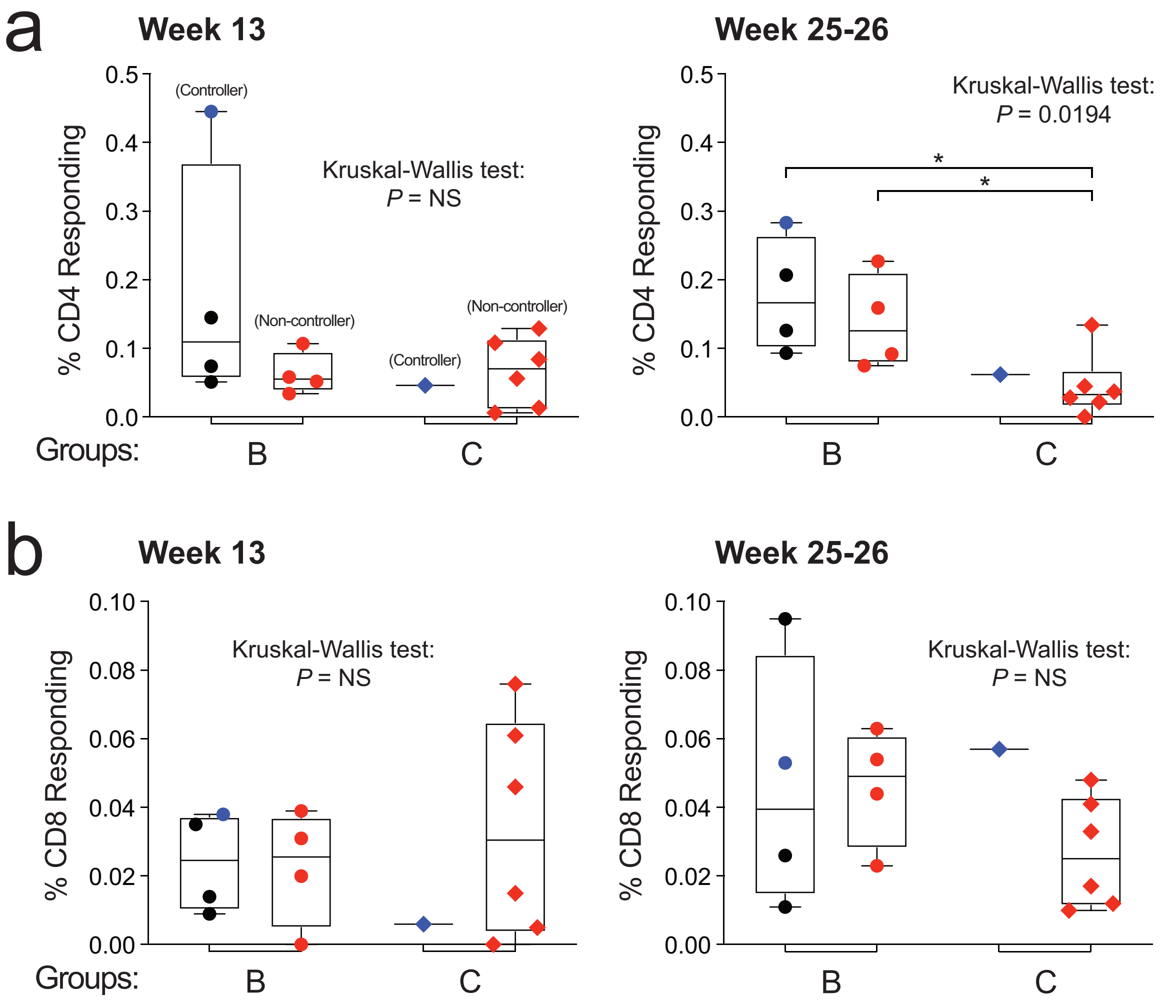

Supplementary Figure S6. Comparison of SIV Gag-specific T-cell responses in RhCMV SIV-vaccinated RM prior to SIV infection. Data presented include CD4 (a) and CD8 (b) T-cell responses for controllers (labeled in black and *Mamu-B*17*-positive RM labeled in blue) and non-controllers (labeled in red). Responses were evaluated by flow cytometric ICS for TNF and/or IFN-γ expression in gated CD4 or CD8 T-cells after stimulating PBMC with overlapping peptides for SIV Gag protein. Overall differences were determined by Kruskal-Wallis test (*P* values shown; NS, not significant) and the uncorrected Dunn's test was used to determine the significance of pairwise differences (*, $P < 0.05$).
